# Supplementary material for: Constitutive activation of two-component systems reveals regulatory network interactions in Streptococcus agalactiae
Source: Nat Commun. 2024 Oct 24;15:9175. doi: 10.1038/s41467-024-53439-3 (PMC11502775; doi:10.1038/s41467-024-53439-3)
Supplement: Supplementary file 1 — Supplementary Information [file 41467_2024_53439_MOESM1_ESM.pdf]

Supplementary Figure 1: Growth phenotype of the HK$^+$ collection

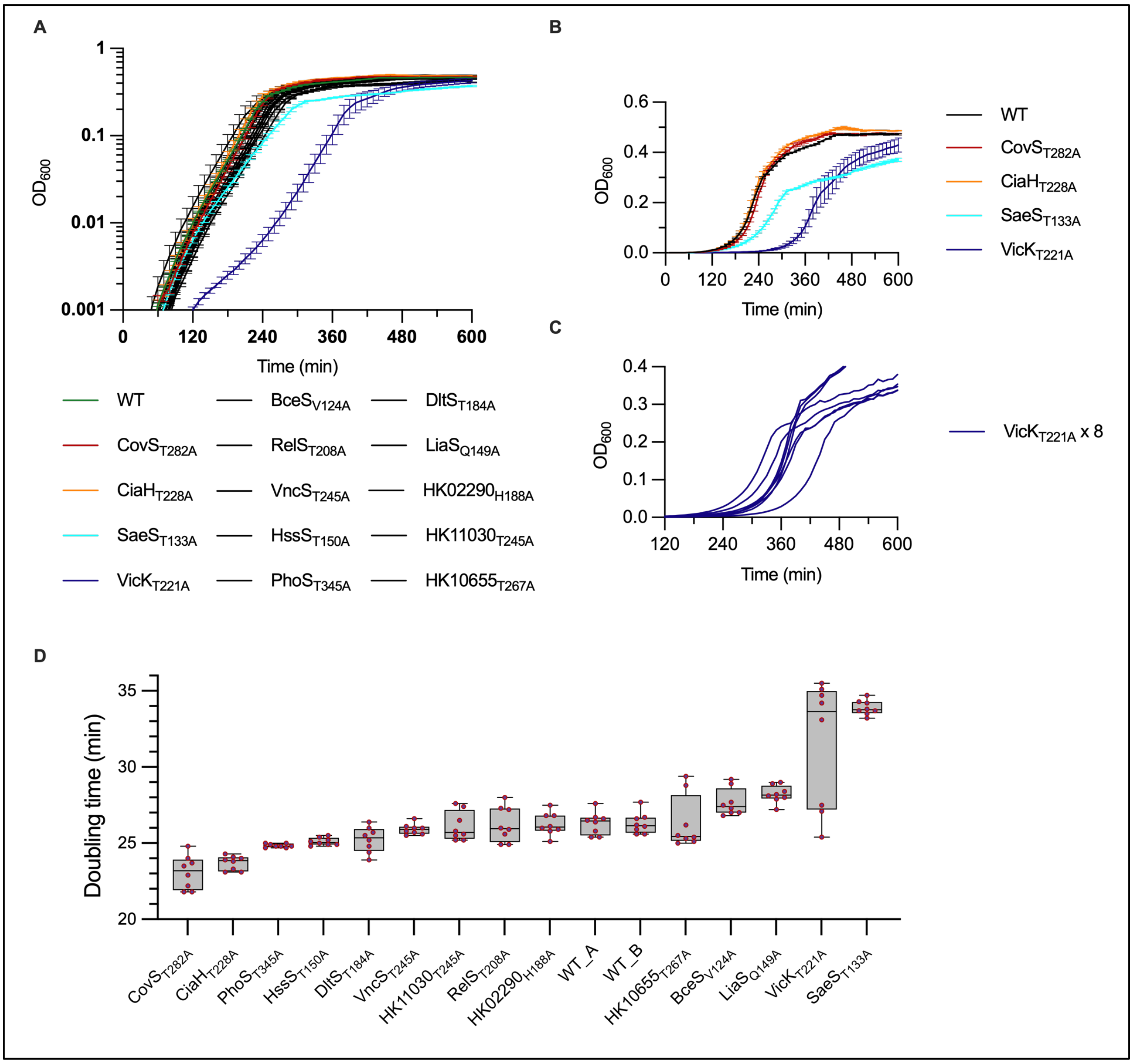

A. Growth curves of the HK$^+$ collection. Cultures were done in rich media (THY) at 37°C in microplate starting from independent isolated colonies. Data represents the mean with SEM for each mutant (n = 8 biological replicate). The control strain and the four HK$^+$ mutants with significant fitness effects (Fig. 1) are highlighted (WT: green; CovS$_{T282A}$: red; CiaH$_{T228A}$: yellow; SaeS$_{T133A}$: light blue; VicK$_{T221A}$: dark blue).

B. Same data as in panel A in non-logarithmic scale highlighting the four mutants with significant phenotype.

C. Individual growth curves of the 8 biological replicate of the VicK$_{T221A}$ mutants.

D. Doubling time in exponential growth phase. Malthusian non-linear fitting ($r^2 > 0.99$) between OD_{600} 0.001 and 0.25 (panel A) were used to infer doubling time. Boxes represent the inter-quartile distance with median (horizontal lines), and the whiskers highlight minimal and maximal value (n = 8 biological replicate). Source data are provided as a Source Data file.

Supplementary Figure 2: Significant differential gene expression in the HK$^+$ collection

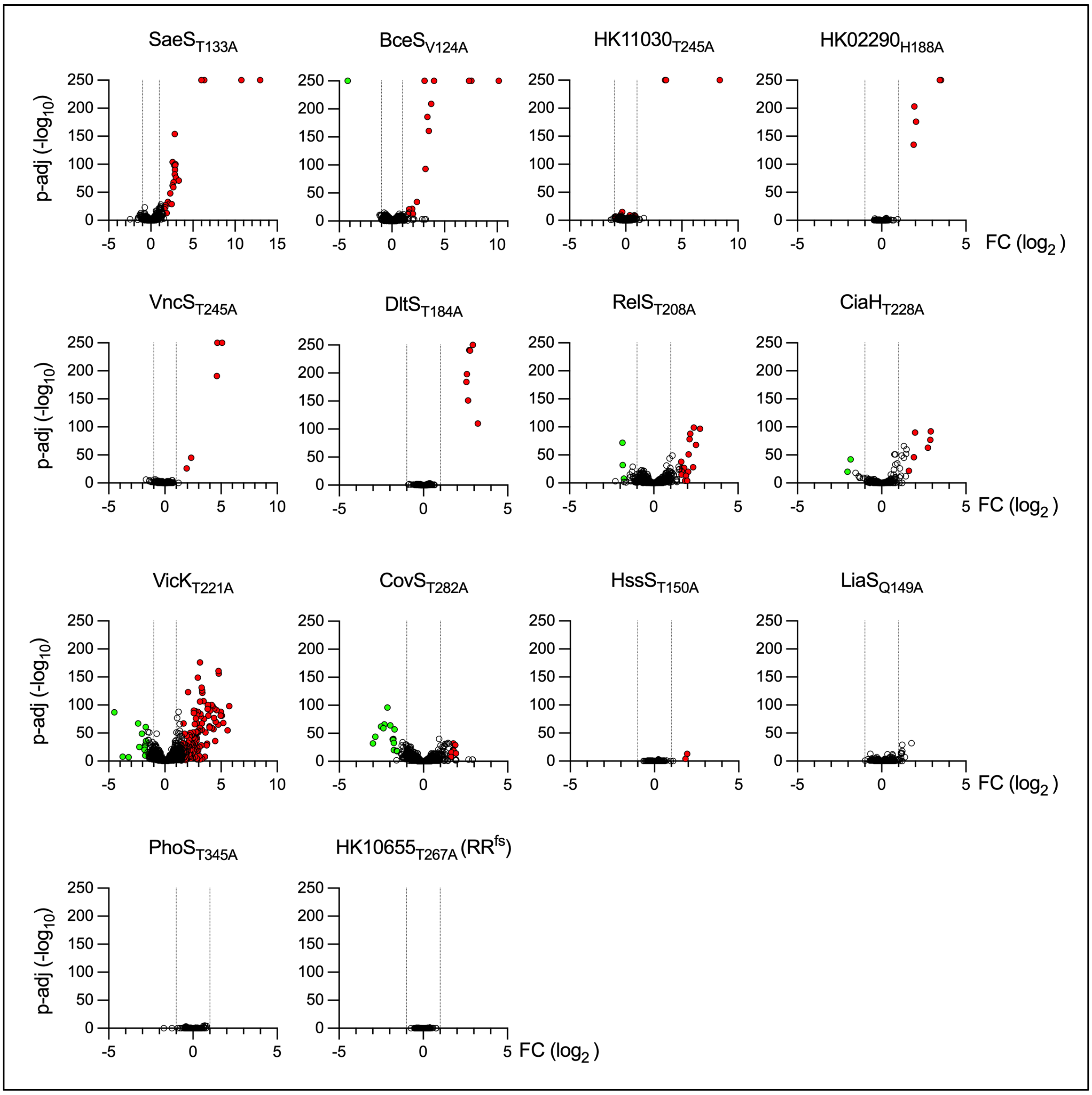

Volcano plot of significant differential gene expression by RNA-seq in exponential growth phase at 37°C in THY for each HK$^+$ mutant against the WT strain (n = 3 biological replicate). Coloured dots represent significantly activated (red) and repressed (green) genes (|FC| > 3, p-adj < 10^{-4} after Benjamini and Hochberg multiple comparison), respectively. RNA-seq data and statistics are provided in Supplementary Data 4D.

Supplementary Figure 3: Activated chromosomal loci by HK^{+}

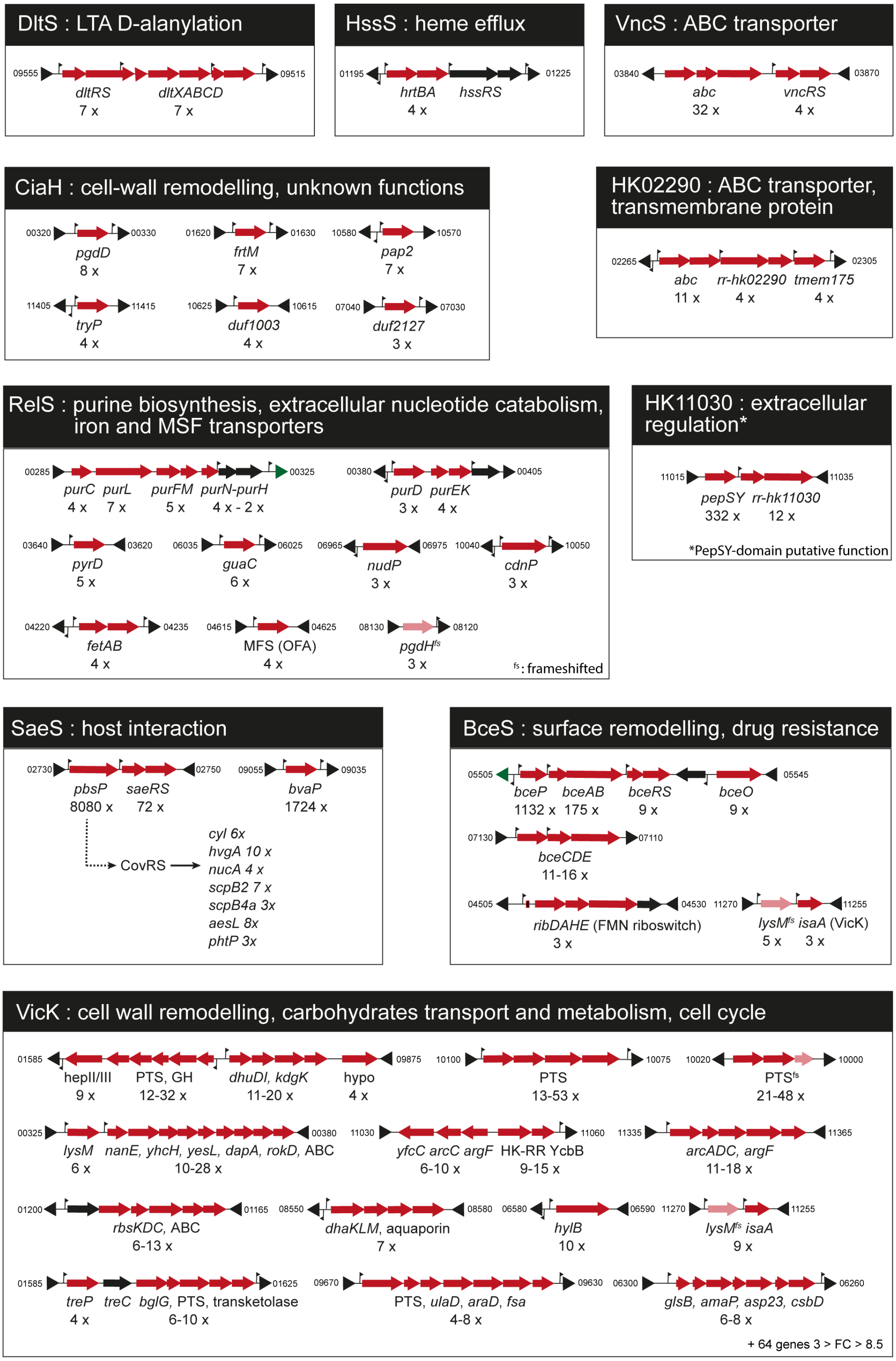

Fold changes determined by RNA-seq are indicated below the activated genes (red arrows). Transcriptional start sites identified by genome wide TSS mapping are represented by vertical flags. NCBI genes ID bordering the loci are shown in a shortened form (e.g.: 11015 = BQ8897_RS11015). Frameshifted genes in the WT are marked (fs). Source data are provided in Supplementary Data 4F.

Supplementary Figure 4: PbsP and BvaP contribute to SaeS$_{T133A}$ fitness defect

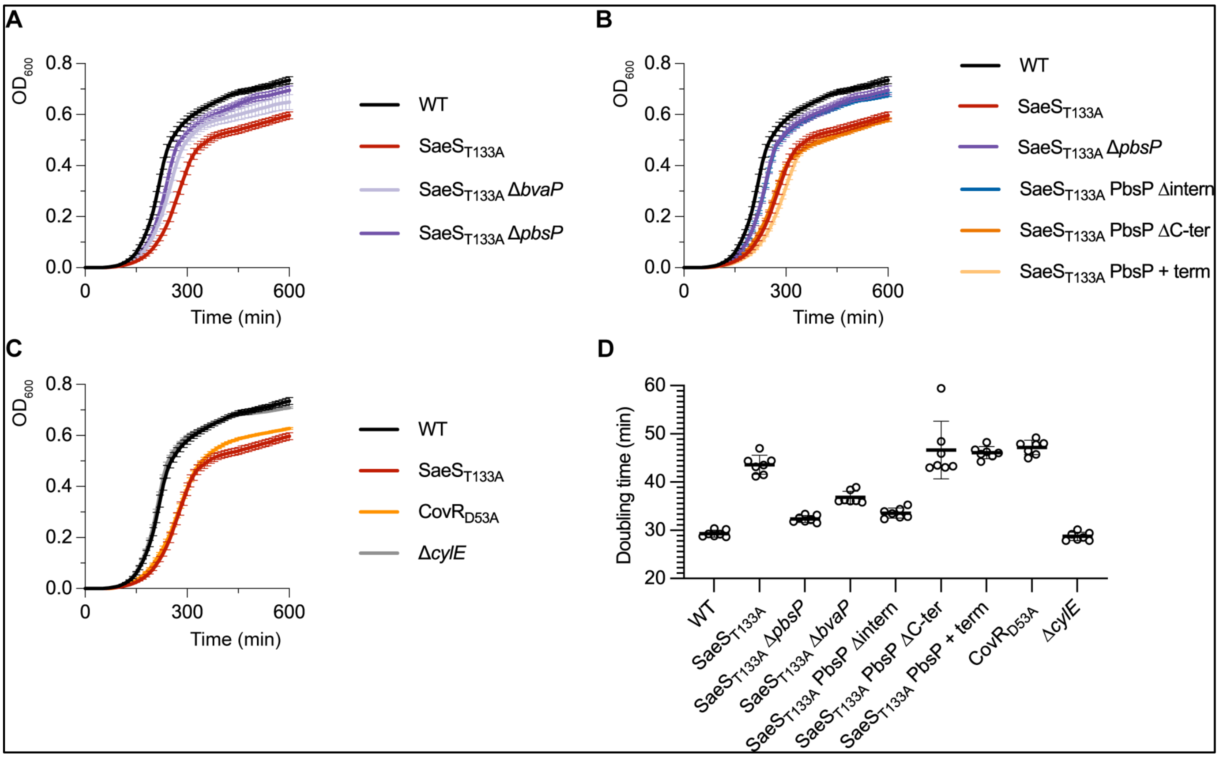

A. Growth curves of the WT (black), SaeS$_{T133A}$ activated mutant (red), and the double SaeS$_{T133A}$ Δ*pbsP* (dark blue) and SaeS$_{T133A}$ Δ*bvaP* mutants (light blue). Data represent the mean +/- SD of a single experiment with pre-cultures inoculated with independent isolated colonies (n = 8 biological replicate).

B. Same experiment with SaeS$_{T133A}$ PbsP Δintern (blue), SaeS$_{T133A}$ PbsP ΔC-ter (orange), and SaeS$_{T133A}$ + *pbsP* term (light yellow).

C. Same experiment with CovR$_{D53A}$ (yellow) and Δ*cylE* (grey).

D. Corresponding doubling time in exponential growth phase. Malthusian non-linear fitting ($r^2 > 0.99$) between OD_{600} 0.02 and 0.4 were used to infer doubling time. Dots represent individual biological replicate (n = 8) and lines represent mean +/- SD. Source data are provided as a Source Data file.

Supplementary Figure 5: Drug susceptibilities of BceS$_{V124A}$

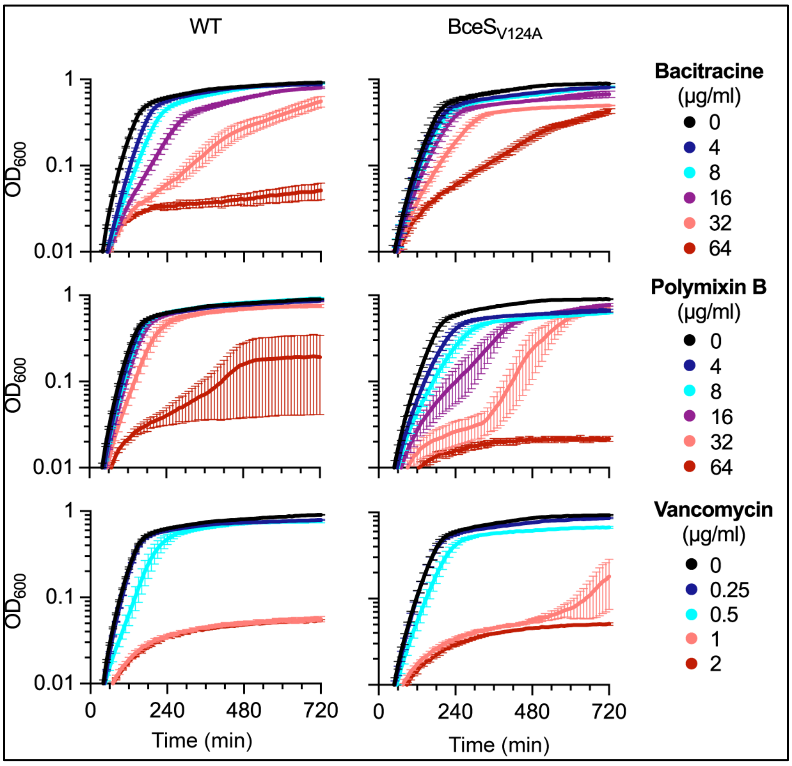

Growth curves of the WT (left panels) and activated BceS$_{V124A}$ mutant (right panels) in presence of increasing concentration of bacitracin, polymyxin B, or vancomycin. Black lines represent the growth in THY in absence of drugs, and coloured lines represent the growth in presence of increasing concentration of a drug (dark blue to red). Curves are mean and SEM of biological replicate (n = 3). Source data are provided as a Source Data file.
