# Supplementary material for: Constitutive activation of two-component systems reveals regulatory network interactions in Streptococcus agalactiae
Source: Nat Commun. 2024 Oct 24;15:9175. doi: 10.1038/s41467-024-53439-3 (PMC11502775; doi:10.1038/s41467-024-53439-3)
Supplement: Supplementary file 3 — Description of Additional Supplementary Files [file 41467_2024_53439_MOESM3_ESM.pdf]

Description of Additional Supplementary Files:

Supplementary Data 1: TCS-encoding genes in the hypervirulent BM110 strain.

Supplementary Data 2: List of strains with sequenced genome.

Supplementary Data 3: Antibiotic susceptibility.

Supplementary Data 4: Transcriptome analysis of the HK+ collection.

4A: All results - RNA-seq normalized count - Mean of biological triplicate.

4B: All results - Statistical analysis.

4C: Genes excluded from the analysis (rRNA, transposase, mobile genetic elements, no expression).

4D: Differentially expressed genes (DEG) - threshold p-adj < 0.05

4E: TCS feedback loops.

4F: Activated genes in the HK+ collection (FC > 3, p-adj < 0.0001)

4G: Repressed genes in the HK+ collection (FC < -3, p-adj < 0.0001)

4H: Activated regulon in the HK+ collection (-3 > FC > 3, p-adj < 0.0001)

Supplementary Data 5: Oligonucleotides.

Supplementary Data 6: Vector and mutant construction.
