# Supplementary material for: Constitutive activation of two-component systems reveals regulatory network interactions in Streptococcus agalactiae
Source: Nat Commun. 2024 Oct 24;15:9175. doi: 10.1038/s41467-024-53439-3 (PMC11502775; doi:10.1038/s41467-024-53439-3)
Supplement: Supplementary file 10 — Reporting Summary [file 41467_2024_53439_MOESM10_ESM.pdf]

nature portfolio

Corresponding author(s): Arnaud FIRON

Last updated by author(s): Sep 17, 2024

Reporting Summary

Nature Portfolio wishes to improve the reproducibility of the work that we publish. This form provides structure for consistency and transparency in reporting. For further information on Nature Portfolio policies, see our Editorial Policies and the Editorial Policy Checklist.

Statistics

For all statistical analyses, confirm that the following items are present in the figure legend, table legend, main text, or Methods section.

n/a	Confirmed	
☐	☒	The exact sample size (*n*) for each experimental group/condition, given as a discrete number and unit of measurement
☐	☒	A statement on whether measurements were taken from distinct samples or whether the same sample was measured repeatedly
☐	☒	The statistical test(s) used AND whether they are one- or two-sided<br>*Only common tests should be described solely by name; describe more complex techniques in the Methods section.*
☒	☐	A description of all covariates tested
☐	☒	A description of any assumptions or corrections, such as tests of normality and adjustment for multiple comparisons
☐	☒	A full description of the statistical parameters including central tendency (e.g. means) or other basic estimates (e.g. regression coefficient) AND variation (e.g. standard deviation) or associated estimates of uncertainty (e.g. confidence intervals)
☐	☒	For null hypothesis testing, the test statistic (e.g. *F*, *t*, *r*) with confidence intervals, effect sizes, degrees of freedom and *P* value noted<br>*Give P values as exact values whenever suitable.*
☒	☐	For Bayesian analysis, information on the choice of priors and Markov chain Monte Carlo settings
☒	☐	For hierarchical and complex designs, identification of the appropriate level for tests and full reporting of outcomes
☒	☐	Estimates of effect sizes (e.g. Cohen's *d*, Pearson's *r*), indicating how they were calculated

Our web collection on statistics for biologists contains articles on many of the points above.

Software and code

Policy information about availability of computer code

Data collection	No software was used for data collection
Data analysis	Geneious Prime v2019.2.3<br>cutadapt v2.10<br>Bowtie v2.5.1<br>featureCounts, v2.0.0<br>R v4.0.5<br>DESeq2 v1.30.1<br>Cytoscape v3.9.1

For manuscripts utilizing custom algorithms or software that are central to the research but not yet described in published literature, software must be made available to editors and reviewers. We strongly encourage code deposition in a community repository (e.g. GitHub). See the Nature Portfolio guidelines for submitting code & software for further information.

Data

Policy information about availability of data

All manuscripts must include a data availability statement. This statement should provide the following information, where applicable:

- Accession codes, unique identifiers, or web links for publicly available datasets
- A description of any restrictions on data availability
- For clinical datasets or third party data, please ensure that the statement adheres to our policy

The RNA-seq data generated in this study have been deposited in the Gene Expression Omnibus database under accession code GSE261394 [https://www.ncbi.nlm.nih.gov/geo/query/acc.cgi?acc=GSE261394]. Additional RNA-seq and ChIP-seq data already reported are available under accession code GSE261394 [https://www.ncbi.nlm.nih.gov/geo/query/acc.cgi?acc=GSE158049]. Source data are provided with this paper.

Research involving human participants, their data, or biological material

Policy information about studies with human participants or human data. See also policy information about sex, gender (identity/presentation), and sexual orientation and race, ethnicity and racism.

Reporting on sex and gender	The study does not involve human participants.
Reporting on race, ethnicity, or other socially relevant groupings	The study does not involve human participants.
Population characteristics	The study does not involve human participants.
Recruitment	The study does not involve human participants.
Ethics oversight	The study does not involve human participants.

Note that full information on the approval of the study protocol must also be provided in the manuscript.

Field-specific reporting

Please select the one below that is the best fit for your research. If you are not sure, read the appropriate sections before making your selection.

☒ Life sciences ☐ Behavioural & social sciences ☐ Ecological, evolutionary & environmental sciences

For a reference copy of the document with all sections, see nature.com/documents/nr-reporting-summary-flat.pdf

Life sciences study design

All studies must disclose on these points even when the disclosure is negative.

Sample size	No sample-size calculation was performed. Independent experiments were performed using biological replicas, in accordance with standards in the field.
Data exclusions	No data were excluded
Replication	Experiments were repeated independantly (biological replicates). For RNA-seq experiments, three independent replicate were done on different days to account for batch effect.
Randomization	Not relevant for this study. Experiments did not require experimental groups.
Blinding	Not relevant for this study. Experiments did not require group allocation.

Reporting for specific materials, systems and methods

We require information from authors about some types of materials, experimental systems and methods used in many studies. Here, indicate whether each material, system or method listed is relevant to your study. If you are not sure if a list item applies to your research, read the appropriate section before selecting a response.

Materials & experimental systems

n/a	Involved in the study
☐	☒ Antibodies
☒	☐ Eukaryotic cell lines
☒	☐ Palaeontology and archaeology
☒	☐ Animals and other organisms
☒	☐ Clinical data
☒	☐ Dual use research of concern
☒	☐ Plants

Methods

n/a	Involved in the study
☒	☐ ChIP-seq
☒	☐ Flow cytometry
☒	☐ MRI-based neuroimaging

Antibodies

Antibodies used

ANTI-FLAG antibody produced in rabbit - polyclonal - unconjugated - affinity isolated - Millipore / Sigma-Aldrich F7425 (dilution: 1/500).
Secondary antibody: IRDye 800CW Goat anti-Rabbit IgG, Li-Cor 926-32211 (dilution:1/5000).

Validation

Commercial affinity isolated antibodies against FLAG epitope: https://www.sigmaaldrich.com/FR/fr/product/sigma/f7425

Plants

Seed stocks

The study does not use plants.

Novel plant genotypes

The study does not use plants.

Authentication

The study does not use plants.
